# Brain functional connectivity changes in children that differ in impulsivity temperamental trait

**DOI:** 10.3389/fnbeh.2014.00156

**Published:** 2014-05-06

**Authors:** Alberto Inuggi, Ernesto Sanz-Arigita, Carmen González-Salinas, Ana V. Valero-García, Jose M. García-Santos, Luis J. Fuentes

**Affiliations:** ^1^Basque Center for Cognition, Brain and LanguageSan Sebastián, Spain; ^2^Neuroimage Department, CITA-Alzheimer FoundationSan Sebastian, Spain; ^3^Radiology and Image Analysis Centre, VU Medical CentreAmsterdam, Netherlands; ^4^Departamento de Psicología Evolutiva y de la Educación, University of MurciaMurcia, Spain; ^5^Servicio de Radiología, Hospital Morales MeseguerMurcia, Spain; ^6^Departamento de Psicología Básica y Metodología, University of MurciaMurcia, Spain; ^7^Regional Campus of International Excellence “Campus Mare Nostrum”, University of MurciaMurcia, Spain

**Keywords:** rs-MRI, functional connectivity, default mode network, impulsivity trait, MRI

## Abstract

Impulsivity is a core personality trait forming part of normal behavior and contributing to adaptive functioning. However, in typically developing children, altered patterns of impulsivity constitute a risk factor for the development of behavioral problems. Since both pathological and non-pathological states are commonly characterized by continuous transitions, we used a correlative approach to investigate the potential link between personality and brain dynamics. We related brain functional connectivity of typically developing children, measured with magnetic resonance imaging at rest, with their impulsivity scores obtained from a questionnaire completed by their parents. We first looked for areas within the default mode network (DMN) whose functional connectivity might be modulated by trait impulsivity. Then, we calculated the functional connectivity among these regions and the rest of the brain in order to assess if impulsivity trait altered their relationships. We found two DMN clusters located at the posterior cingulate cortex and the right angular gyrus which were negatively correlated with impulsivity scores. The whole-brain correlation analysis revealed the classic network of correlating and anti-correlating areas with respect to the DMN. The impulsivity trait modulated such pattern showing that the canonical anti-phasic relation between DMN and action-related network was reduced in high impulsive children. These results represent the first evidence that the impulsivity, measured as personality trait assessed through parents' report, exerts a modulatory influence over the functional connectivity of resting state brain networks in typically developing children. The present study goes further to connect developmental approaches, mainly based on data collected through the use of questionnaires, and behavioral neuroscience, interested in how differences in brain structure and functions reflect in differences in behavior.

## Introduction

Impulsivity is a core personality trait that entails difficulties to inhibit responding, novelty seeking, and the inability to delay rewarding (Barratt, 1994). While impulsivity as a trait forms part of normal behavior and contributes to adaptive functioning, altered patterns of impulsivity have been shown to be a major component of diverse behavioral disorders. For instance, features of impulsive behavior in response inhibition are associated with people diagnosed with attention deficit hyperactive disorder (ADHD) (Nigg, 2000), but also with those diagnosed with borderline (Hazlett et al., 2005) and antisocial personality disorders (for a review, see Glenn and Raine, 2011), and those with risk of substance use disorders (Perry and Carroll, 2008; Verdejo-García et al., 2008; Moreno et al., 2012) commonly observed also during adolescence (Stautz and Cooper, 2013). Even in typically developing children and adolescents, impulsiveness has been pointed as a risk factor for the development of behavioral problems, and alongside with low inhibitory control and high negative emotionality proneness, it plays a role in a number of externalizing problems (Krueger et al., 1996; Lengua et al., 1998; Olson et al., 1999; Lemery et al., 2002; Eisenberg et al., 2005; Sterzer and Stadler, 2009). A set of brain areas has been associated with poor impulse control and executive dysfunction. The bulk of evidence involves the frontal lobe in impulsive behavior, mainly the orbitofrontal cortex (Horn et al., 2003; Berlin et al., 2004; Völlm et al., 2004; Antonucci et al., 2006) and the ventromedial frontal lobe (Narayan et al., 2007), although the inferior frontal cortex has also been included due to its role in response inhibition (Aron et al., 2004). However, some other brain regions such as the amygdala (Blair, 2007), the temporal lobe (Dolan et al., 2002), the angular gyrus (Soderstrom et al., 2000) and the posterior cingulate cortex (Tiihonen et al., 2008) have also been described as related to impulsive and antisocial behavior (Glenn and Raine, 2011). Recent neuroimaging and lesion studies are revealing details of brain activity of extreme relevance to better understand the brain abnormalities underling different aspects of impulsive behavior exhibited by non-violent individuals but also by very aggressive offenders, with or without psychiatric diagnoses (Shannon et al., 2011). Yet it is an empirical question whether these brain areas associated with impulsiveness in clinical population can also discriminate individual differences in impulsivity in normal population. As Hahn et al. (2012) suggest, investigating a potential link between personality and brain dynamics using a correlative approach appears important, as pathological and non-pathological states are commonly characterized by continuous transitions rather than categorical boundaries. In normal population, a few studies have identified adult impulsivity personality trait associated with areas of sensitiveness to reward, and areas of attentional and executive control. More specifically, the ventral striatum and the orbitofrontal cortex have been identified as key regions related to impulsivity (Tanaka et al., 2004; Hahn et al., 2012). However, to our knowledge, even fewer studies have looked at the neural correlates of impulsive behavior in typically developing children with no psychiatric diagnoses. We hypothesize that some of the areas that are involved in severe impulsive behavior might be differentially activated in children that exhibit higher levels of trait impulsivity compared to those showing lower levels of impulsivity.

To examine this question we assessed impulsivity trait in a sample of typically developing children aged 8–12 years through the temperament in middle childhood questionnaire (TMCQ) combined with the analysis of the brain basal activity patterns by means of resting-state fMRI (functional magnetic resonance imaging). The TMCQ (parent-report version; Simonds and Rothbart, 2006) was developed under Rothbart and Derryberry's framework of temperament (1981). From this approach, temperament is defined as constitutionally based individual differences in reactivity and self-regulation. In this context, constitutional refers to the relatively enduring biological make-up of the individual, influenced by heredity, maturation, and experience. Reactivity refers to the arousability of emotional, motor, and attentional responses, assessed by threshold, latency, intensity, time to peak intensity, and recovery time of reactions. Finally, self-regulation includes processes such as attention that can serve to modulate reactivity (Rothbart and Derryberry, 1981; Posner and Rothbart, 2009). In this framework, TMCQ impulsivity Scale, shortly defined as “speed of response initiation” (Simonds and Rothbart, 2006), covers difficulties to inhibit responding (i.e., s/he can't help touching things without getting permission), unreflecting responses (i.e., s/he tends to say the first thing that comes to mind, without stopping to think about it), and inability to delay gratification (i.e., s/he grabs what s/he wants), a taxonomy that clearly mimics that of Evenden's (1999) in animal studies. Previous factor analyses proved that this scale forms part of the Surgency/Extraversion factor (Putnam and Rothbart, 2006). Extraverts are described as emotionally positive, reward oriented, active, and outgoing, seeking stimulation or novelty. These characteristics have been also compiled in other renowned personality frameworks (Eysenck and Eysenck, 1985; Gray, 1987; McCrae and Costa, 1987). From a developmental point of view, impulsivity has a tendency to decrease with age during childhood and throughout adolescence (Avila et al., 2004; Galvan et al., 2007; Steinberg et al., 2008) as a consequence of synaptic pruning and the continued myelination of prefrontal brain regions that result in improved connectivity among cortical areas and between cortical and subcortical areas (Steinberg et al., 2008). Yet, individual differences not explained by age can be found in children and adolescents, but it is still an empirical question whether the same patterns of connectivity are associated with equivalent temperament traits in children compared to adults. In fact, previous research such as the work by Whittle et al. (2014) found, opposite to their expectation based on adult findings, that a reduced orbitofrontal cortex (OFC) volume was associated with a lower temperamental Effortful Control in adolescents.

In order to understand if individual differences in impulsivity scores, as measured through parents' report, might be mediated by specific brain activity patterns, we correlated the individual scores of the TMCQ test with the subjects' brain activity recorded at rest. Also known as resting-state activity, the brain functional signature product of this condition corresponds to a specific cognitive state characterized by the absence of salient stimuli or cognitive goal-driven behavior. In the last decade, research on the brain's resting-state activity has gained interest because it is thought to reflect basal, intrinsic brain activity (Raichle and Snyder, 2007; Buckner et al., 2008; Power et al., 2010; Sandrone, 2012, for reviews). When measured by means of functional magnetic resonance imaging (fMRI), resting-state activity results in robust and sustained spontaneous activity reflected by low-frequency (<0.1 Hz) correlated blood oxygenation level dependent (BOLD) signal fluctuations in cortical and subcortical brain regions. These activity patterns can be analyzed with a variety of functional connectivity techniques (Calhoun et al., 2001; Beckmann et al., 2005). Interestingly, coherent activity patterns between anatomically independent brain regions have been interpreted as the bases of interregional communications (Biswal et al., 1995). Therefore brain regions sharing a coherent activity pattern during the duration of the resting-state condition are regarded as intrinsic connectivity networks, also called resting state networks (RSNs) (Damoiseaux et al., 2006). While some sources of coherent BOLD signal can be identified as acquisition artifacts and noise sources like movement or respiratory-related signals, most RSNs show a close spatial mapping with known functional systems activated in task-based fMRI paradigms (Deco and Corbetta, 2011 for a review).

The study of one RSN in particular, the default mode network (DMN), has become a pivotal topic in experimental and clinical neuroscience due to its activity pattern in relation to that of the rest of networks (Fox and Raichle, 2007; Sandrone, 2012). DMN activity is stronger when subjects are not involved in any specific task (resting-state condition) and therefore it is anti-correlated to those action-related RSN (e.g., the attentional, salience, executive and motor networks) whose activity increases during task execution (Raichle et al., 2001; Fox et al., 2005; Margulies et al., 2007; Di Martino et al., 2008; Kelly et al., 2008; Uddin et al., 2009). DMN physiological activity is known to exert a great influence over the action-related networks (Uddin et al., 2009) and the reduction of such anti-phasic relation has been repeatedly associated to performance decrease (Weissman et al., 2006; Kelly et al., 2008), physiological aging (Sambataro et al., 2010) and cognitive and behavioral disorders (Buckner et al., 2008).

Although each RSN is defined as a functionally homogeneous aggregate of anatomically independent brain areas, such unique relation is just an approximation and several functional sub-units may be present. This is particularly true for the DMN, where functional sub-units can be discriminated in terms of their specific activity pattern (Sestieri et al., 2011), their relevance within the local or global connectivity network (Fransson and Marrelec, 2008) or the influence they exert on the other resting-state networks (Uddin et al., 2009). In order to take into account the presence of such functional sub-units within each RSNs, a voxel-based approach was thus used. To study the relation between basal brain activity and impulsivity, we employed a two levels approach: firstly we explored which regions of the DMN might be differentially activated in children that exhibit higher levels of trait impulsivity compared to those showing lower levels of impulsivity. Then, we calculated the functional connectivity among these regions and the rest of the brain in order to assess if impulsivity trait alters the relationships between DMN and action-related networks.

## Materials and methods

### Subjects

Twenty-four healthy children participated in this study. All were Spanish White children, with Spanish as their first language, recruited from a school located in middle-class area in Murcia, Spain. The majority of children (20/24) lived with both biological parents, whereas the remaining 4 lived with only one biological parent. Family income reports in euros per month were as follows: 12.5% (3/24) reported between 1000 and 1400; 50% (12/24) reported between 1401 and 2000; 29.2% (7/24) reported between 2001 and 3000; and 8.3% (2/24) reported more than 3000. Parental education was as follows: 8.3% of the mothers (2) and 12.5 of the fathers (3) reported 6 years of schooling; 66.7% of the mothers (16) and 58.3% of the fathers (14) reported 12 years of schooling; and the remaining had a college or university degree. Data from five children were excluded because of imaging artifacts induced by excessive motion and/or orthodontic prostheses. An informed consent was obtained from children's parents that were present to each part of the experiment. The experiment was approved by the ethical committee of the Hospital Morales Meseguer where the present study was conducted.

### Measurement of children's temperament

Parents informed of their children's temperament through a Spanish version of the temperament in middle childhood questionnaire (TMCQ; Simonds and Rothbart, 2006, unpublished Spanish version, University of Murcia). It was composed of 158 items or statements describing children's characteristics. Parents had to indicate in which extent each statement properly described his/her child's behavior within the previous 6 months. The scale ranged from 1 (almost always untrue) to 5 (almost always true), with an additional option of “Not applicable.” TMCQ assesses 17 lower-order facets of temperament. Score in each scale was calculated dividing the total by the number of items receiving a numerical response. For the purpose of this study, impulsivity scale was selected. Cronbach's alpha for this scale reached 0.85, with an item-test correlation mean of *r* = 0.59 (range from 0.44 to 0.72). Teachers gave the questionnaire in an enclosed envelope to the children so that parents could fill it out at home. Once completed, parents returned the questionnaire to the teachers. A researcher was available at school and by phone to attend any question concerning the questionnaire.

### MRI scan acquisition

During the acquisition of the MRI functional session, the participant children were instructed to lie as still as possible with their eyes closed and not focus on a specific activity or thought. To ensure the child's comfort, a parent sat in silence next to the child in the room during the scanning session. Hearing was protected using earplugs and motion was minimized using soft pads fitted over the ears. Functional and anatomical images were acquired in the same session on a GE 1.5 T HDX scanner (GE, USA). For the resting-state sequence, 200 echo-planar imaging (EPI) images sensitive to blood-oxygenation-dependent-level (BOLD) contrast were acquired with the following sequence parameters: 24 slices; repetition time (TR) 1.888 ms; echo time (TE) 55 ms; voxel size 4 × 4 × 4 mm; field-of-view (FOV) was 25.6 × 25.6 cm with a 64 × 64 matrix, and a flip angle of 90°; scan duration 6:30 min. A high-resolution T1-weighted scan using a 3D FSPGR BRAVO sequence was acquired to enable co-registration of functional data to an anatomical template. The sequence parameters were: TR 12.4 ms; TE 5.2-15 ms; voxel size 1 × 1 × 1 mm; flip angle 12°; 142 axial slices. In addition, a one-volume EPI covering the whole brain (38 slices) with the same acquisition parameters of the sequence used for resting state was acquired in order to improve the co-registration accuracy.

### Preprocessing and denoising

EPI image preprocessing was carried out using FSL (Version 4.19), FMRIB's Software Library (www.fmrib.ox.ac.uk/fsl) (Smith et al., 2004; Woolrich et al., 2009), involving non-brain removal using Brain Extraction Tool, BET, (Smith, 2002); motion correction with MCFLIRT (Jenkinson et al., 2002); high-pass temporal filtering using Gaussian-weighted least-squares straight line fitting (width = 100 s); spatial smoothing using a Gaussian kernel with full-width half-maximum 5 mm. Children images displaying motion artifacts with a maximum displacement over 1 mm were excluded from the study. Due to the importance of controlling the influence of motion artifacts common in children studies, we further denoised the pre-processed EPI images by means of a single-subject independent components analysis (ICA; Beckmann and Smith, 2004; Beckmann et al., 2005). ICA is a mathematical technique that allows the separation of linearly mixed sources of data. Applied to fMRI analysis, ICA is capable of dividing the BOLD signal into different homogeneous elements or components. Each of these components represents a coherent spatio-temporal pattern and can correspond to either temporally correlated activity patterns of spatially independent brain areas, reflecting brain functional networks, or coherent noise sources like movement, heartbeat or breathing artifacts. By visually analyzing the spatial patterns and temporal characteristics of the resulting spatio-temporal components we removed those related to residual motion (function RegFilt, FSL library; Foerde et al., 2006; de Bie et al., 2012).

### Group template

Considering that using an adult-based anatomical template would have introduced a severe bias into pediatric imaging data by introducing anatomical co-registration errors (Hoeksma et al., 2005), we created a custom pediatric template through a procedure previously used to analyze resting state data of children (de Bie et al., 2012). First, we performed a 12-degree of freedom affine registration of each subject's T1 image to the MNI152 T1 brain template, using FLIRT (FMRIB version 5.92, Oxford, UK). Then the mean inverse transformation of all the subjects was calculated and applied to the MNI152 template (resolution 4 mm isotropic) in order to create the pediatric custom template of our children participants. Second, we co-registered the individual EPI images to such common template following a three-steps procedure: (a) co-registration of the individual EPI images to their corresponding one-volume whole-brain EPI images, (b) co-registration of whole-brain EPI images to their corresponding T1, and c) co-registration of the individual T1 to the pediatric template (resampled at 4 × 4 × 4 mm). Finally the resulting multi-step co-registration matrix was then applied to the individual EPI images.

### Independent components analysis (ICA) and dual regression

After denoising and coregistration of the individual EPI sequences, we performed a group temporal concatenation independent components analysis (TC-ICA; Beckmann and Smith, 2004), as implemented in FSL (MELODIC, version 3.10), producing a set of independent components common to the whole group. Every independent component consists of a brain map, with an associated temporal course of the brain's activity, which represents a functionally connected network common to the entire group. In order to allow group-level statistical comparison, the equivalent of each group component was individuated in every individual subject through the dual regression analysis (Filippini et al., 2009; Zuo et al., 2010). Such procedure consists of (1) a spatial regression using the ICA maps as regressors, followed by (2) a temporal regression using the representative time courses as regressors producing subjects' maps containing voxel-wise regression coefficients of each component's time course. The maps of the individual subjects corresponding to the DMN were merged in a 4D image for correlation analyses. In addition, a group mask of all DMN-related RSN was created from the thresholded *z*-stat (*Z* ≥ 2.3) images defined by group MELODIC analysis (Beckmann et al., 2005).

### DMN correlation with impulsivity

In order to define the relationship between children's impulsivity and their connectivity within the DMN, we designed a group-level general linear model (GLM) including two columns with gender and demeaned age values, to correct for their effect, and a column with demeaned impulsivity scores. The model was analyzed using permutation-based non-parametric testing independently for each individual DMN component, limiting the analysis to the previously calculated DMN group mask (number of permutations = 5000; Randomize, FSL function library; Nichols and Holmes, 2002). The resulting maps where thresholded by threshold-free cluster enhancement (TFCE; Smith and Nichols, 2009) using a significance threshold of *p* < 0.05 and then corrected for multiple comparisons using Family-Wise Error criteria.

### Seed-based functional connectivity (SBFC) analysis

**Figure 1:**
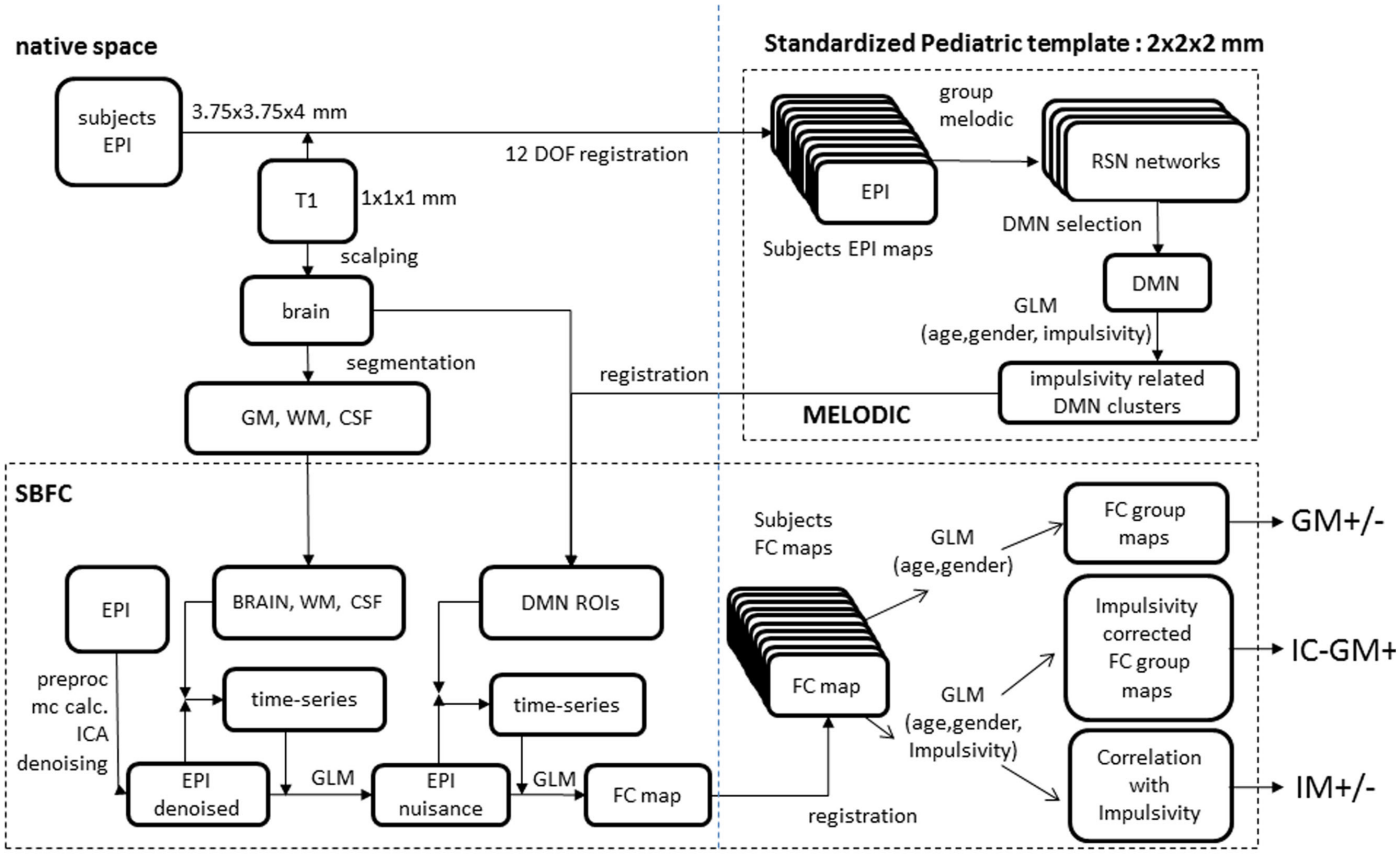
**Analyses scheme**. (Upper right) melodic analysis. (lower pane) seed-based functional connectivity analysis. Details of EPI to custom template registration, involving anatomical T1 and one-volume whole-brain EPI images were omitted for simplicity.

To assess the functional connectivity between impulsivity-related areas within the DMN and the rest of the brain, a seed-based functional connectivity analysis was performed. At the subject level we carried out a two-step regression analysis. In the first step, mean time-series of white matter, CSF and whole brain volumes were calculated from the denoised individual data and their effect was regressed out. Then, regions of interest (ROIs) that resulted in the previous analysis were co-registered to each subject's space through a 12 DOF linear affine transformation implemented in FLIRT and their mean time-series were then calculated. Replicating a previous study investigating the functional connectivity of multiple brain ROIs (Margulies et al., 2007; Di Martino et al., 2008), all these time-series were inserted as regressors in a single GLM and orthogonalized according to the Gram Schmidt process as implemented in FEAT. The outputs of this analysis are subject-level maps of all those voxels correlating and anti-correlating with each investigated ROI. At the group level, two different GLM were tested. The first GLM included only age and gender regressors and produced two functional connectivity group maps including those voxels with functional connectivity correlated (GM+) or anti-correlated (GM-) with seeds' ones. Then a second GLM was used incorporating also the individual impulsivity scores to (a) evaluate if such scores might modulate the correlating/anti-correlating relation between the two seeds and the rest of the brain (impulsivity-corrected group maps: IC-GM+ and IC-GM−), and (b) to attribute a sign to such modulation, by calculating a direct relation between impulsivity and the functional connectivity of the seeds region (IM+ and IM−). Group-level analyses were carried out using a mixed-effects model (FLAME) as implemented in FSL. Corrections for multiple comparisons were carried out at the cluster-level using Gaussian random field theory (*Z* > 2.3; cluster significance: *p* < 0.05, corrected). The analysis process is summarized in Figure 1.

## Results

At the time of testing, the mean age of the analyzed 19 subjects (9 girls, 10 boys) was 9.9 years (range 8.1–12.1; *SD* 1.4). Participants underwent the Kaufman brief intelligence test (KBIT). Mean IQ was 108 (range 89–122). Mean impulsivity score was 3.3 (range 2.36–4; *SD* 0.44). To test whether such rather low variability might be different from those expected in typically developing children, we compared the present impulsivity scores with those of a larger group of same age (*N* = 76, mean impulsivity = 2.9; *SD* = 0.60). We tested the homogeneity of the variances with a Bartlett test (Chi-Square = 2.53, *p* = 0.11). This non-significant result indicates that the impulsivity of the group examined is similar to that expected in larger population of typically developing children and therefore our results have not been biased by the randomness of the children recruitment process. The relative and absolute mean displacements within the MRI scanner were 0.11 ± 0.038 mm (range 0.06–0.21 mm) and 0.34 ± 0.15 mm (range 0.12–0.66 mm) respectively.

### Identification of group RSN

**Figure 2:**
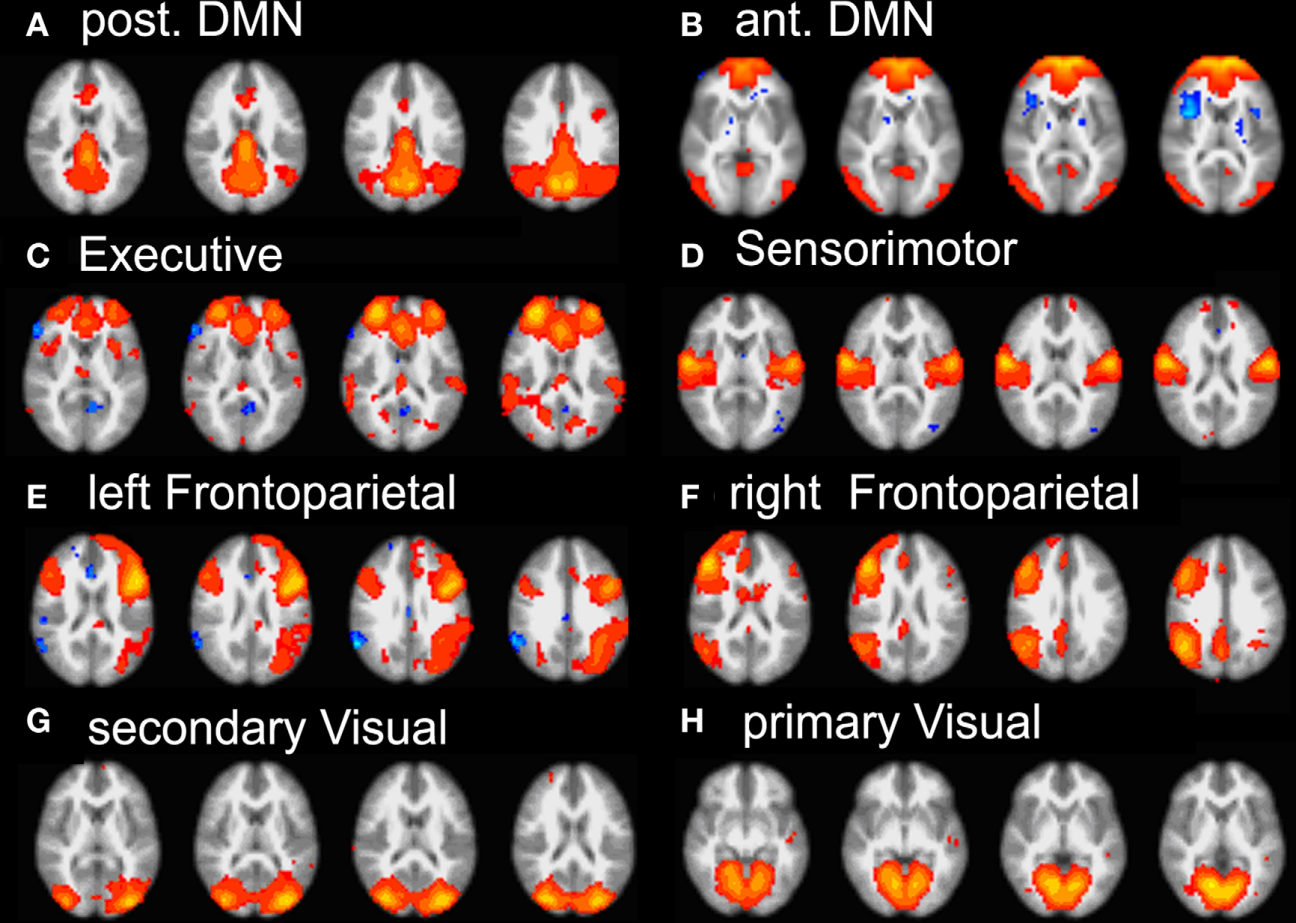
**Summary of the eight RSN revealed by group-melodic analysis. (A)** posterior DMN, **(B)** anterior DMN, **(C)** executive, **(D)** somatosensory, **(E)** left and **(F)** right frontoparietal, **(G)** secondary and **(H)** primary visual.

Group ICA of subjects' data revealed 22 independent components; 8 were selected by visual inspection as anatomically consistent areas potentially reflecting functionally relevant RSNs. The remaining 14 components were discarded as largely reflecting head motion, physiological noise, or image fluctuations in cerebral spinal fluid. The classic DMN was found in the form of two distinct and partially overlapping components: (i) a posterior DMN centered on posterior cingulate/precuneus subsystem and extending to the angular and supramarginal gyri (SMG) and medial temporal lobe (MTL), (ii) an anterior DMN centered on ventromedial prefrontal cortex (vmPFC). Additionally, six further networks were found: right and left fronto-parietal, executive (anterior salience), primary visual (in striate and extra-striate regions), secondary visual areas and the sensory motor network RSN maps (see Figure 2).

### Dual regression: DMN correlations with impulsivity

**Figure 3:**
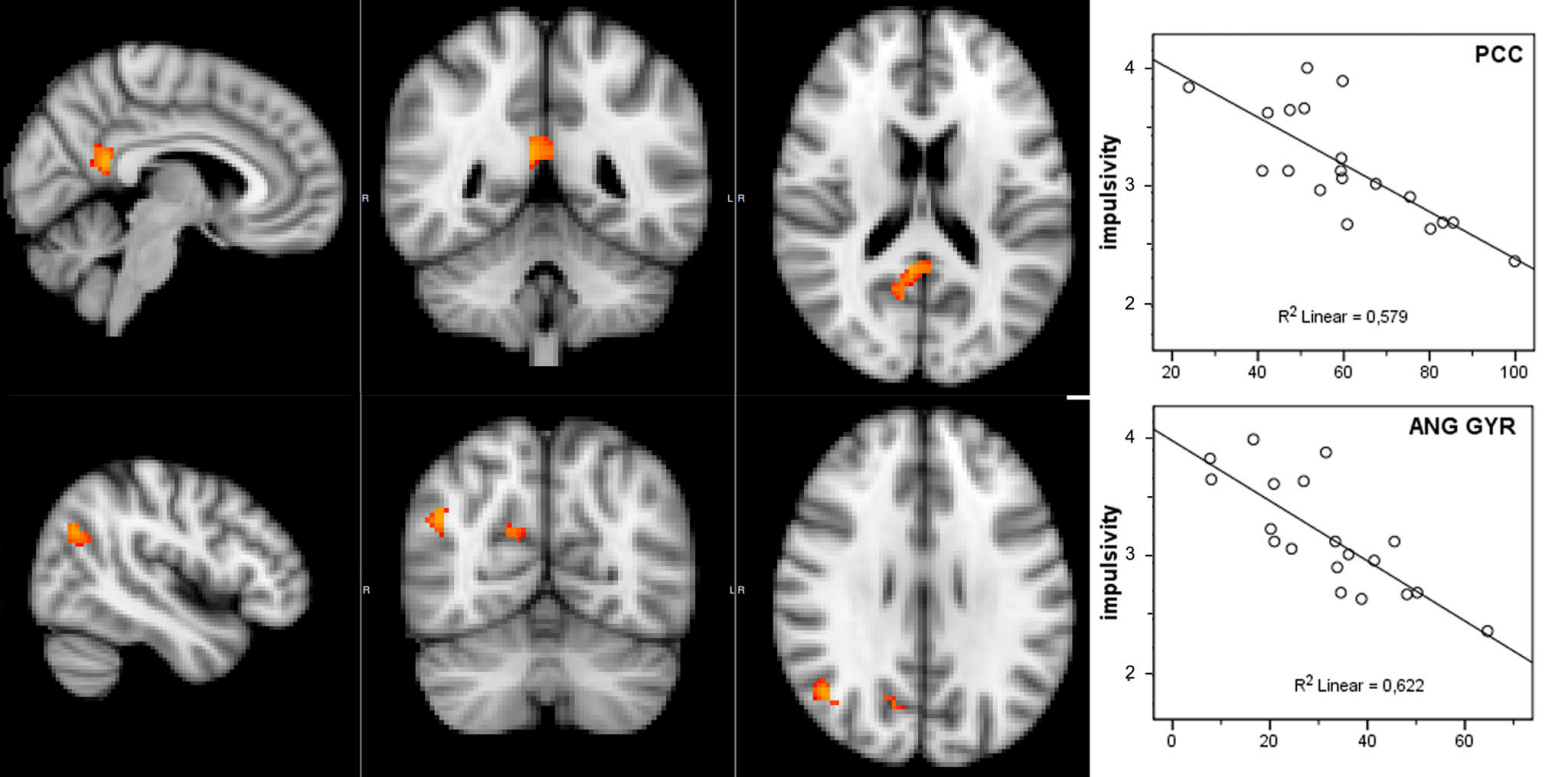
**Negative correlation between functional connectivity within the DMN and participant's impulsivity scores: (left) correlation overlaid over anatomical template, (right) scatter plot of functional connectivity *PE* values and impulsivity scores**.

Considering that the correlation analysis was performed over both RSNs representing the DMN, only those voxels that after the TFCE correction showed a *p*-value below 0.025 were reported as significant. A negative correlation between impulsivity and functional connectivity was found in two posterior DMN clusters, one located in the posterior cingulate cortex (PCC) and one in the right angular gyrus (AG). Correlating voxels are displayed in Figure 3, foci coordinates and *p*-values are summarized in Table 1.

**Table 1:** **Negative correlation between Impulsivity Scale and DMN**.

**Region**	***p*-value**	***z*-score**	**MNI space**		
			***X***	***Y***	***Z***
PCC	0.0048	5.18	6	−46	16
AG	0.012	4.01	46	−58	28

*Cluster p-value and z-score and position, in MNI coordinates, of the voxel showing the maximum z-score value*.

*PCC, posterior cingulate cortex; AG, angular gyrus*.

### Seed-based functional connectivity

**Figure 4:**
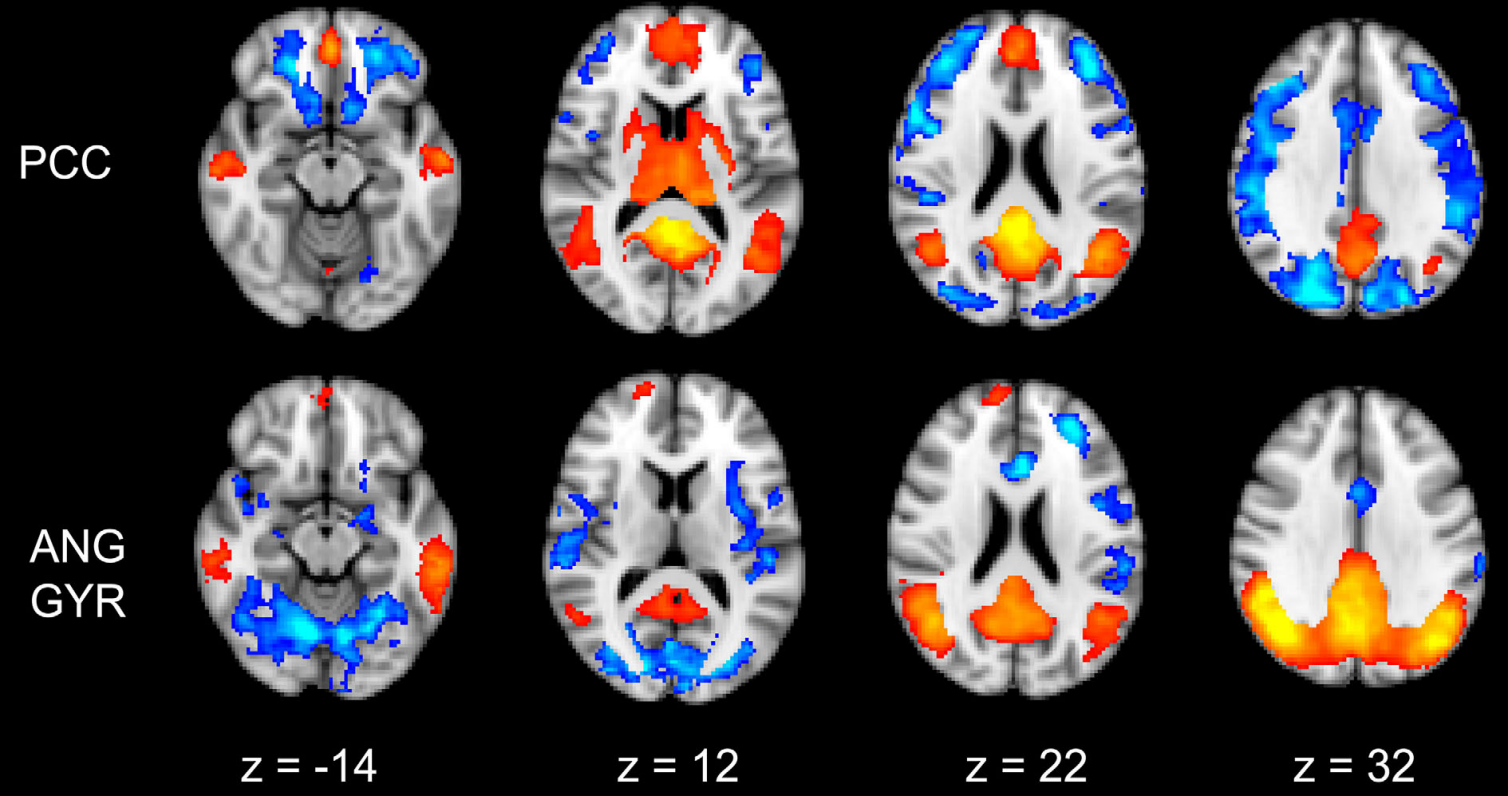
**Functional connectivity group maps (GM+, GM−) of the two DMN ROIs (PCC, AG) that resulted negatively correlated with impulsivity score**. In yellow-red color scale are displayed the correlating voxel (the GM+ contrast), in light blue-blue color scale the anti-correlating ones (the GM− contrast). The axial slice number is displayed below each column.

**Figure 5:**
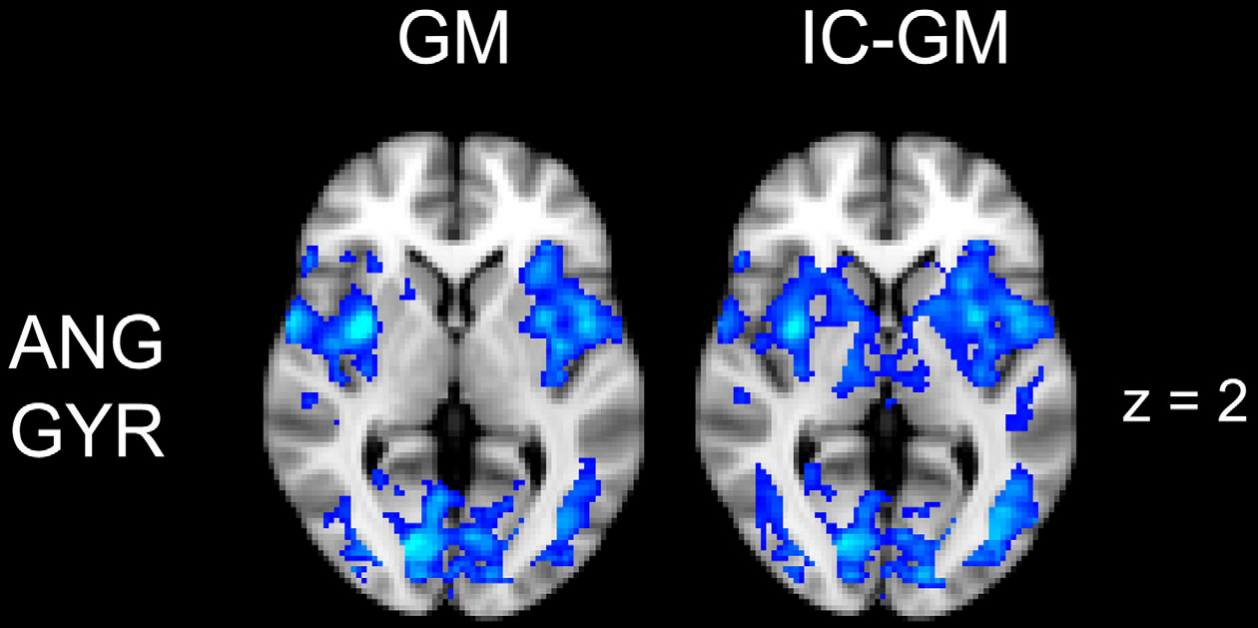
**Effect of impulsivity score in functional connectivity group maps**. In blue-light color scale are displayed anti-correlating. In the left column is displayed the negative functional connectivity GM- of angular gyrus with the rest of the brain. In the right column are displayed the connectivity maps of the same AG cluster after inserting the participants impulsivity Scale scores in the GLM (IC-GM).

**Figure 6:**
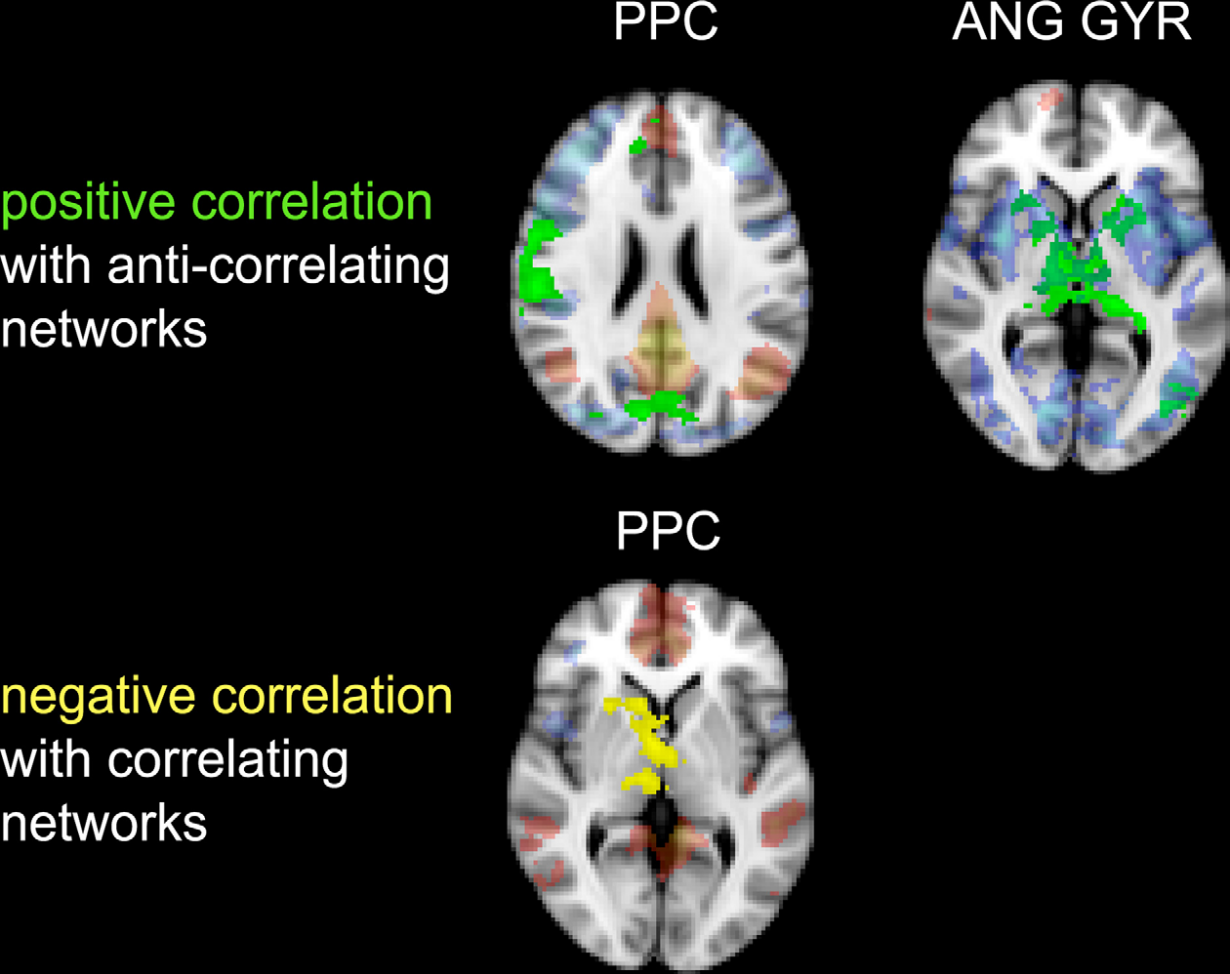
**Correlation between impulsivity and functional connectivity of the two seed clusters PCC and AG**. (**Upper**) positive correlation (IM+), drawn in green, with voxels belonging to the anti-correlating network (IC-GM−). (**Lower**) negative correlation (IM−), drawn in yellow, with voxels belonging to the correlating network (IC-GM+). In semi-transparent mode are displayed the correlating (red) and anti-correlating (blue) voxel of IC-GM+ and IC-GM−.

The functional connectivity between the two DMN clusters (PCC and AG) and the rest of the brain was explored with a seed-based functional connectivity analysis (SBFC) in two steps. First, we explored the group's pattern of correlating and anti-correlating networks (GM+/−) specific to PCC and AG (Figure 4; Table 2). Then, by incorporating into the GLM also the individual impulsivity scores, we explored the effect of children impulsivity over such pattern (IC-GM+/−, Figure 5; Table 3) and the direct positive (IM+) and negative (IM+/−) relation between impulsivity and functional connectivity maps of the two cluster (Figure 6; Table 4). The resulting patterns of the full-brain seed-based connectivity analysis for both PCC and AG regions and their relation to impulsivity are described below.

**Table 2:** **Group maps**.

**PCC**	**Corr. sign**	**Cluster *p*-value**	**Region**	**Side**	***z*-score**	**FSL-MNI space**		
						***X***	***Y***	***Z***
	POS	<0.00001	PCC	–	8.77	2	−48	16
			Precuneus	–	7.09	2	−58	16
			Lat occipital cx. / AG	L	6.15	−42	−62	18
			Lat occipital cx. / AG	R	4.22	44	−58	18
		<0.00001	vmPFC	–	5.74	−4	50	−10
			Paracing g./ACC	L	5.3	−10	40	−2
			Paracing g./SFG	–	4.63	−2	50	22
		<0.00001	AG	R	4.63	50	−52	18
			MTG anterior	R		56	−10	18
		Striatal areas manually identified						
			Putamen	L	4.14	−28	−14	10
			Thalamus	L	4.69	−4	12	12
			Thalamus	L	4.70	−10	30	12
			Thalamus	R	4.19	8	−18	12
			Caudate	L	3.26	−10	10	10
			Putamen	R	3.07	24	0	12
			Caudate	R	3.04	10	4	12
	NEG	<0.00001	Lat occipital cx	R	5.46	26	−80	34
			Lat occipital cx	L	5.03	−24	−82	34
			MFG/DLPFC	R	4.86	38	42	22
			MFG/DLPFC	L	4.82	−40	42	30
			Inf prec sulcus/IFJ/IFG	R	4.47	46	6	24
			Inf prec sulcus/IFJ/IFG	L	3.5	−42	4	24
			SMG	L	4.24	−54	−38	32
			SMG	R	4.03	46	−28	26
			OFC	R	4.88	20	38	−16
			OFC	L	4.63	−16	38	−18
			ITG	L	4.42	−46	−34	−18

*AG, angular gyrus, SMG, supramarginal gyrus; OFC, orbitofrontal cortex; SFG, superior frontal gurus; MFG, middle frontal gyrus; IFG, inferior frontal gyrus; vmPFC, ventromedial prefrontal cortex; ACC, anterior cingulate cortex; IFJ, inferior frontal junction; ITL, inferior temporal lobe; MTL, middle temporal lobe; PCC, posterior cingulate cortex; cx, cortex; g, gyrus*.

**Table 3:** **Additional areas functionally connected with angular gyrus after correction for impulsivity**.

**ANG GYR**	**Corr. sign**	**Region**	**Side**	***z*-score**	**FSL-MNI space**		
					***X***	***Y***	***Z***
	NEG	Putamen	L	3.44	−28	10	2
		Thalamus	R	3.4	16	−16	4
		Putamen	R	3.38	22	18	0
		Thalamus	L	3.22	−10	−18	4

**Table 4:** **Correlations between seed-based functional connectivity and impulsivity scale**.

**ROI**	**Corr. sign**	**Cluster *p*-value**	**Region**	**Side**	***z*-score**	**FSL-MNI space**		
						***X***	***Y***	***Z***
PCC	POS	0.0041	SMG anterior	R	3.83	58	−26	26
			Inf. Precentral g.		3.53	58	0	26
			Postcentral g.		3.54	62	−10	22
			Parietal Operc cx.		3.22	48	−26	28
		0.0165	Cuneal cx.	R	3.88	14	−80	30
			Lateral occipital cx.		3.12	30	−86	20
			Cuneal cx.	L	2.83	−6	74	24
		0.0366	Para−cingulate g.	R	4.01	8	44	20
			Frontal pole		2.99	8	58	20
			ACC	L	3.13	−2	46	10
	NEG	0.0019	Caudate	R	3.62	10	16	0
			Thalamus		3.46	6	−2	4
			Thalamus		2.9	8	−18	2
			Putamen		3.06	26	16	6
			Thalamus	L	3.21	−2	−8	4
ANG GYR	POS	0.00011	Thalamus	R	3.28	12	−12	2
			Thalamus	L	3.27	−18	−30	2
			Thalamus	L	3.25	−10	−4	2
			Putamen	L	3.25	−24	16	0
			Putamen	R	2.89	22	16	0
			Pallidum	L	3.27	−14	2	0
			Pallidum	R	2.91	20	4	0
		0.000553	Lat inf occipital	L	3.86	−38	−76	−4
			Lingual g.	L	3.12	−2	−72	−8

*SMG, supramarginal gyrus; ACC, anterior cingulate cortex*.

### PCC cluster

The PCC resulted functionally correlated (GM+/−) with a group of brain regions mostly overlapping with the extent of the DMN (cingulate/paracingulate cortices, posterior cingulate/precuneus, parietal and temporal lobe), with the addition of striatal (caudate, putamen) and thalamic regions. PCC was functionally anti-correlated with parietal (supramarginal gyrus, superior parietal cortex) and frontal regions (inferior frontal gyrus, inferior frontal junction, dorsolateral prefrontal cortex), the anterior cingulate cortex and the insula (Figure 4). No differences were found after adding the impulsivity scores to the analysis (IC-GM+/−). The correlation analysis between PCC functional connectivity and impulsivity scores (IM+/−) revealed that in the impulsive subjects, PCC was more negatively correlated with right caudate, putamen and thalamus, which belong to the PCC-correlating network, and more positively correlated with paracingulate cortex, and three regions included in the PCC-anti-correlating network: the cuneal cortex, the inferior precentral sulcus and the supramarginal gyrus (SMG) (Table 4).

### Angular gyrus cluster

The functional connectivity analysis revealed correlated activity of AG with the classical posterior DMN pattern embracing the posterior cingulate/precuneus cortex, AG, SMG and medial temporal gyrus. The AG anti-correlated network included occipito-temporal, limbic and cingulate areas (Figure 4). When the impulsivity scores were inserted into the GLM (IC-GM+/−), also striatal (pallidum and putamen) and thalamic areas resulted anti-correlated with the AG cluster (Figure 5). Impulsivity scores (IM) resulted positively correlated with bilateral striatal and thalamic regions, as well as medio-temporal and occipital cortices, this last one included in the anti-correlated network of this cluster (see Figure 6).

## Discussion

Though impulsive behavior has been related to specific brain networks in animals and human adults, little is known about the relation between impulsivity as personality trait and brain network activity in normal human development. Here we report that in 8–12 years-old children higher impulsivity trait is related to lower resting state brain activity within the default mode network (DMN). In particular, we found two brain regions of the DMN, the PCC and the AG, whose connectivity is negatively correlated to children's level of impulsivity. We further report the correlating and anti-correlating connectivity pattern of PCC and their relationship to impulsivity. We found that the more impulsive children were, the more negatively correlated PCC was to the regions composing its correlating network, (caudate, putamen and thalamus) and more positively correlated to part of its anti-correlated network (SMG, inferior precentral sulcus, cuneal cortex). The connectivity pattern of the AG was also altered: as the children showed higher impulsivity AG was more positively correlated to the striatal and thalamic areas as well as to constituents of its anti-correlated network. To our knowledge, this is the first time the impulsivity as personality trait has been related to different resting state functional brain networks in typically developing children.

### DMN in 8–12 years-old children

In our group of children the DMN was found segregated in two partly overlapping but differentiable sub-networks consisting of a posterior DMN centered on posterior cingulate/precuneus and an anterior one centered on ventral middle prefrontal cortex (vmPFC). Although generally considered as a single functional entity, the split of the DMN into anterior and posterior sub-networks has been found in a number of studies. In particular, the subdivision is common in those that tested children, prompting the hypothesis that such subdivision might represent a stage in the incomplete maturation of children networks connections (de Bie et al., 2012). This particularity has been also reported in recent studies investigating a very large sample (*N* = 603; Allen et al., 2011), which has led some authors to suggest instead that the two sub-networks may account for some of the variations in the specific spatial distributions or functional specialization of the default mode network (Buckner et al., 2008; Harrison et al., 2008). These two hypotheses are complementary if we consider that those different modules might start as segregated sub-networks, integrating them in the proper DMN through a process of brain maturation. Accordingly, these differences could be more easily observed in children, where the integration has not yet been completed, or in studies where the huge sample offer a much higher statistical sensitivity to the subtle differences present in adults. Importantly, our own data point out to the functional specialization of parts of the DMN, as they single out its posterior parts, PCC and AG, as being specifically related to impulsivity trait and further modulating the activity of other brain networks.

### Disconnection within the DMN

The resulting connectivity maps indicate a relative functional disconnection within the DMN in those children with higher impulsivity score in specific regions of the posterior cingulate cortex (PCC) and right angular gyrus (AG). This was confirmed by repeating the analysis to include the whole brain (either restricting to gray matter or not), which resulted in the original significant PCC and AG clusters with reduced but still significant *p*-values. Both regions are of critical importance for the functioning of the DMN. PCC is considered as the connecting center for information arriving from different functional networks and its distribution to the rest of the DMN (Hagmann et al., 2008), and its activity is involved in a wide range of cognitive tasks. Of particular interest are those functional connections with frontoparietal areas belonging to the attentional orienting network, possibly reflecting the PCC's role in maintaining a broad attentional focus. Moreover, electrophysiological studies with non-human primates suggest that PCC signals environmental changes and the need to alter the organism's own behavior (Hayden et al., 2009), leading to the hypothesis that the PCC might be also involved in controlling responses to a rapidly changing environment (Leech et al., 2011; Pearson et al., 2011). The angular gyrus is also implicated in a wide variety of cognitive processes including reasoning and social cognition (see Seghier, 2013, for review). Importantly, neuroimaging investigation of the neural basis of anti-social behavior confirmed the alteration of the activity of these two areas (Raine and Yang, 2006). PCC showed reduced activation in aggressive patients (New et al., 2002), while angular gyrus, which is implicated in a wide range of moral judgment tasks (Greene et al., 2001), resulted hypo-activated in murderers (Raine et al., 1997) and in impulsive violent criminals (Soderstrom et al., 2000). Although these results do not demonstrate a causal relation between the disconnection of these two areas within the DMN and possible anti-social behavior, it is noteworthy that the same areas involved in extreme alterations of behavior appear more functionally disconnected from the other components of the DMN in typically developing children with higher impulsivity scores.

### Disconnection between DMN and the rest of the brain

The analysis of the functional connectivity of PCC and AG with brain regions beyond the DMN widely confirmed previous studies. Posterior cingulate resulted bilaterally positively connected with the components of the DMN (vmPFC, angular gyrus and MTL regions), while angular gyrus resulted connected with posterior regions but also with striatal areas. Both PCC and AG showed a pattern of anti-correlating voxels, which reflects the most specific characteristic of DMN: its functional anti-correlation with action-related networks (Raichle et al., 2001; Fox et al., 2005; Margulies et al., 2007; Di Martino et al., 2008; Kelly et al., 2008; Uddin et al., 2009). Anti-correlation with PCC and AG elements of the DMN was in fact found in areas belonging to the dorsal attention network (supramarginal gyrus, superior parietal cortex, inferior frontal gyrus), which implements the reorienting of attention in response to external stimuli (Corbetta et al., 2008). It was also found in the dorsolateral prefrontal cortex, anterior cingulate, and insula, which belongs to the executive/salience network, which is active in a wide variety of cognitive process involving decision-making in the context of goal-directed behavior (see for review Menon and Uddin, 2010). Finally, anti-correlation with PCC and AG functioning was also found in the inferior frontal junction, an area which connects the inferior precentral sulcus with the inferior frontal one and is involved in working memory, task switching and inhibitory control (Sundermann and Pfleiderer, 2012), and it is likely also deputed to adapt our behavior to a new task environment (Brass et al., 2005).

When we added the impulsivity score as a further regressor in the GLM we could observe some slight differences in AG connectivity, showing further anti-correlated activity with putamen and thalamus bilaterally. We interpreted this finding by suggesting that the impulsivity score might have helped the model to correctly interpreter the BOLD signal fluctuations, allowing stressing the role of striatal and thalamic areas in modulating children impulsivity. These results agree with those from neuroimaging studies that revealed higher activity in ventral striatal nucleus and other connected areas such as the lateral orbitofrontal cortex and insula, when the task favored an immediate reinforce rather than a delayed one (McClure et al., 2004; Tanaka et al., 2004). It is assumed that impulsive people prefer a smaller but immediate reinforce than a bigger but delayed one (Kim and Lee, 2011). Also, striatal dysfunction connectivity, involving mainly the putamen, has been associated with some pathological states that have been characterized as impulsive, like people diagnosed with ADHD (Cao et al., 2009).

The results of the analysis of the correlation between impulsivity scores and PCC/AG functional connectivity are of particular interest. As previously cited, the anti-phasic relation between DMN and action-related networks might predict task performance (Weissman et al., 2006; Kelly et al., 2008) and reduces with healthy ageing (Sambataro et al., 2010) and pathological disease progression (Buckner et al., 2008). If we assume that high values of impulsivity reflect reduced skill in correctly inhibit motor and verbal responses, which might lead to antisocial and rule-breaking behavior, our correlation results are consistent with such assumption. As our results indicate, the DMN of high impulsive children is functionally less connected with canonical rest areas and more related to task-related ones, displaying an opposite trend compared to normal DMN functioning and approaching that observed with ageing and pathological conditions.

### Methodological considerations and limitations

A high influence of gender dimension over temperamental trait has been repeatedly hypothesized (see for review Chapple and Johnson, 2007), To avoid for a possible gender specific effect, the general linear model investigating the relation between impulsivity and functional connectivity within the DMN was corrected for subjects' gender. Similarly, the model was also corrected for age as small variations of functional connectivity are expected among children with age varying from 8 to 12. Although a recent study on adults suggests that resting state analysis does not need to be corrected for age (Weissman-Fogel et al., 2010), a multicentric study with a huge sample revealed gender-related connectivity differences within DMN (Allen et al., 2011). Finally, the analyses were restricted to the RSN group maps in order to reduce our investigation DMN areas common between all subjects.

There is a strong debate over the effect of the global signal regression (GSR) applied in the present study. It has been hypothesized that its tendency to zero-center the correlation might create artefactual anti-correlation among networks (Murphy et al., 2009), as the one observed also in the present study. Even if such split among default mode and action related networks were artifactual, our main findings, a reduced anti-correlation in more impulsive children, goes in the opposite direction of such split and thus the correctness of our consideration should be preserved. Nevertheless, our results are in accordance with the physiological presence of a strong phasic/anti-phasic relation, which represents the competing functions of the involved areas (Fransson, 2005; Margulies et al., 2007; Di Martino et al., 2008; Kelly et al., 2008; Carbonell et al., 2014). This study has some limitations that need consideration. Firstly, the number of subjects investigated prevents a complete generalization of our results. However, the present results are strong in terms of both *p*-values and *z*-scores, which lead to expect further confirmation of the present findings with larger samples of children. Moreover, our correlation should have not been influenced by the low variability of the impulsivity scores of the children we recruited. In fact, this is a sample of typically developing children with ages between 8 and 12 years old; hence it is not surprising that no children scored in the extreme range in impulsivity. Accordingly, similar low variability was found when we analyzed a much bigger samples of children (*N* = 76) with similar socio-demographic characteristics. Secondly, we only examined functional connectivity during resting state; hence future studies examining the effect of impulsivity scores during tasks involving motor inhibition and/or attentional and executive control will be necessary to confirm the areas associated to an impulsive temperament. Nevertheless, this technique allows examining the basal brain activity patterns and proved its potential in predicting the functional relation among different brain areas, relations which noteworthy were then confirmed by fMRI task-related studies (Deco and Corbetta, 2011). Also, the characterization of DMN hubs connectivity promises to be very meaningful as they directly modulate activity in task-positive networks by exerting greater influence on their anti-correlated networks than the other way around (Uddin et al., 2009). Finally, the functional MRI scan did not cover the higher portion of motor areas and portions of the dorsal attention network, which might have prevented us from assessing further relations among impulsive trait and brain functional connectivity.

### Conclusions

Briefly, the present results suggest that differences in brain connectivity are clearly found in children that differ in impulsive behavior according to their parents' report. Temperament traits, considered as individual differences in the tendencies to experience and express emotions and arousal, as well as the ability to regulate those tendencies, are thought to be constitutionally based by heredity and further influenced by maturation and experience (Rothbart and Derryberry, 1981). According to this theory, our results suggest that what parents tell about their children's reactions reflects consistent functional brain differences as a trait. This finding constitutes by itself an important contribution to understand the neural basis of impulsive behavior. Such proneness to impulsiveness is expressed in a reduced anti-correlation between areas belonging to the DMN and to action-related networks, which might represent a possible biological marker of their lower abilities to control their behavior. Future research should reveal whether experience in the sense of learning to adopt appropriate self-regulation strategies, will have an effect not only in the control of impulsive behavior but also in regulating brain networks connectivity. The study of impulsivity along with other temperament dimensions, such as Sensation Seeking, would be of interest for further understanding the development of specific pathologies such as ADHD (Rothbart and Rueda, 2005). The brain networks here reported are also affiliated with impulsivity related neurotransmitter systems, such as dopamine (Dalley and Roiser, 2012). Future research may shed more light on the influence of pharmacotherapy on the network connectivity here reported (see Cole et al., 2013), and the influence of other impulsivity related neurotransmitters such as serotonin and noradrenaline on these network connections (Dalley and Roiser, 2012), in particular in developmental conditions such as ADHD (e.g., Hart et al., 2013). Finally, although some authors have questioned the use of parents' report to study temperament (e.g., Kagan, 1998), our findings clearly demonstrate that the present approach might contribute to our understanding of appropriate methods for the study of individual differences. Our results, along with other studies that have also used parents' and self-reports (e.g., Whittle et al., 2014), support the use of questionnaires as valid methods to measure temperament. This opens an interesting line of further research that connects two different traditions; that of developmental approaches mainly based on data collected through the use of questionnaires, and other based on a behavioral neuroscience perspective, interested in how differences in brain structure and functions reflect in differences in behavior.

## Author contributions

Alberto Inuggi, created the MRI sequence, performed the analysis, interpreted the results, drafted and revised the manuscript. Ernesto Sanz-Arigita, developed the MRI sequence and contributed to analysis, interpretation and drafting of the manuscript. Carmen González-Salinas and Ana V. Valero-García translated the temperament questionnaire in Spanish, recruited the children, interacted with their family and revised the manuscript. Jose M. García-Santos contributed to sequences setup, drafted and revised the manuscript. Luis J. Fuentes designed the experiment, interpreted the data, drafted and revised the manuscript.

### Conflict of interest statement

The authors declare that the research was conducted in the absence of any commercial or financial relationships that could be construed as a potential conflict of interest.

## Abbreviations

ADHD: attention deficit hyperactive disorder
TMCQ: temperament in middle childhood questionnaire
OFC: orbito-frontal cortex
SMG: supramarginal gyrus
MTL: medial temporal lobe
vmPFC: ventromedial prefrontal cortex
AG: angular gyrus
PCC: posterior cingulate cortex
RSN: resting state network
DMN: default mode network
SBFC: seed-based functional connectivity
fMRI: functional magnetic resonance imaging
EPI: Echo-planar imaging
BOLD: blood oxygenation-dependent level
TR: repetition time
TE: echo time
ICA: independent component analysis
GLM: general linear modelç
ROI: region of interest
KBIT: Kaufman brief intelligence test
SD: standard deviations
GSR: global signal regression.
